# Organophosphorus compounds and oximes: a critical review

**DOI:** 10.1007/s00204-020-02797-0

**Published:** 2020-06-06

**Authors:** Franz Worek, Horst Thiermann, Timo Wille

**Affiliations:** Bundeswehr Institute of Pharmacology and Toxicology, Neuherbergstrasse 11, 80937 Munich, Germany

**Keywords:** Organophosphorus compounds, Nerve agents, Pesticides, Acetylcholinesterase, Oximes, Reactivation

## Abstract

Organophosphorus (OP) pesticides and nerve agents still pose a threat to the population. Treatment of OP poisoning is an ongoing challenge and burden for medical services. Standard drug treatment consists of atropine and an oxime as reactivator of OP-inhibited acetylcholinesterase and is virtually unchanged since more than six decades. Established oximes, i.e. pralidoxime, obidoxime, TMB-4, HI-6 and MMB-4, are of insufficient effectiveness in some poisonings and often cover only a limited spectrum of the different nerve agents and pesticides. Moreover, the value of oximes in human OP pesticide poisoning is still disputed. Long-lasting research efforts resulted in the preparation of countless experimental oximes, and more recently non-oxime reactivators, intended to replace or supplement the established and licensed oximes. The progress of this development is slow and none of the novel compounds appears to be suitable for transfer into advanced development or into clinical use. This situation calls for a critical analysis of the value of oximes as mainstay of treatment as well as the potential and limitations of established and novel reactivators. Requirements for a straightforward identification of superior reactivators and their development to licensed drugs need to be addressed as well as options for interim solutions as a chance to improve the therapy of OP poisoning in a foreseeable time frame.

## Introduction

Two contradictory statements “Hitherto, alkylphosphate poisoning has been treated mainly by atropine, but now atropine is replaced by PAM [pralidoxime]” (Namba and Hiraki 1958) and “Based on the current available data on human organophosphate poisoning, oxime was associated with either a null effect or possible harm” (Peter et al. 2006) impressively underline the ongoing discussion on the value of oxime therapy in human poisoning by organophosphorus compound-based (OP) pesticides.

**Fig. 1:**
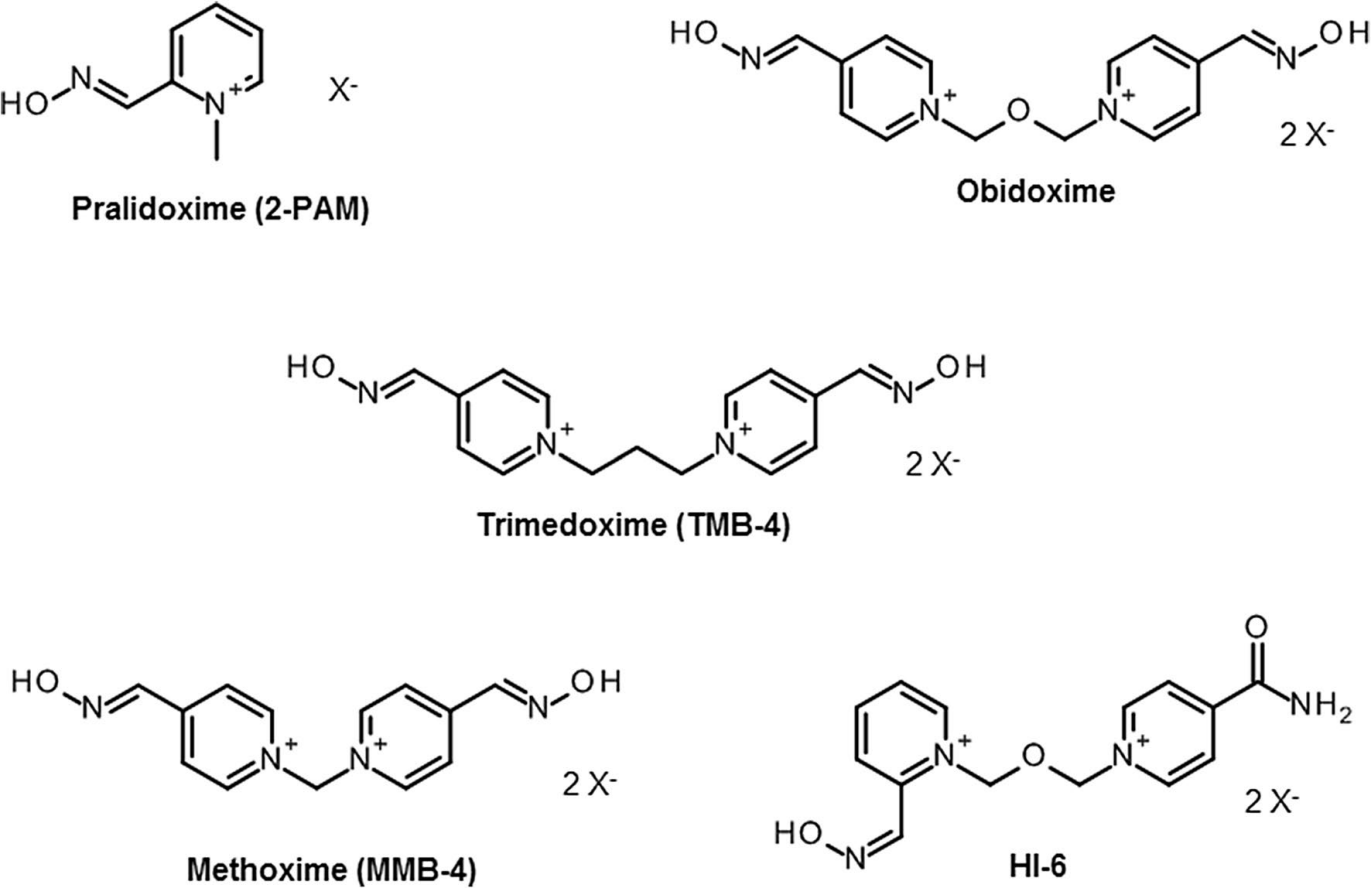
Chemical structure of in-service oximes developed in the 1950s and 1960s. Oximes with different counterions, e.g. pralidoxime chloride (2-PAM) and methanesulfonate (P2S) are available and are used in research and clinical practice

**Fig. 2:**
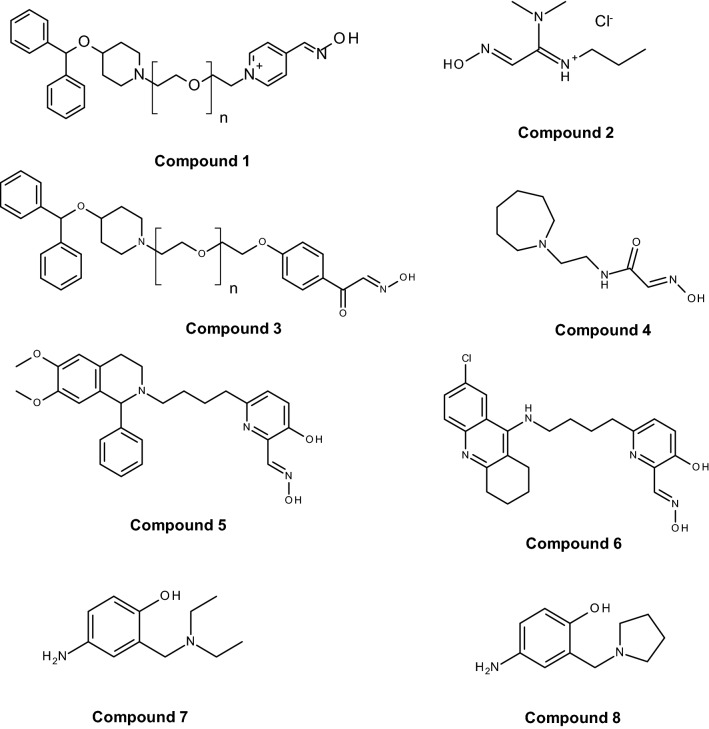
Chemical structure of selected novel oxime and non-oxime reactivators according to de Koning et al. (2011a, b, 2018), Mercey et al. (2011), Radic et al. (2012), Kalisiak et al. (2011), Katz et al. (2015), Santoni et al. (2018)

Since the discovery of pralidoxime (2-PAM; Fig. 1) in 1955 by UK and US research groups (Childs et al. 1955; Wilson and Ginsburg 1955) a countless number of oxime structures were synthetized, initially focused on the identification of an effective antidote against the nerve agent soman and more recently on the development of CNS active reactivators and oximes with a broader spectrum against OP nerve agents and pesticides (Boskovic 1981; Clement 1981; Musilek et al. 2011; Katalinic et al. 2013; Kovarik et al. 2013; Worek and Thiermann 2013; Eyer and Worek 2007; Kuca et al. 2006; Chambers and Meek 2020). Early work was mainly based on the development of charged mono- (pralidoxime-type) and bis-pyridinium (trimedoxime-/obidoxime-type) oximes (Fig. 1), while in the last decade a variety of structural elements were introduced cumulating in uncharged oximes and non-oxime reactivators (Fig. 2) (Bismuth et al. 1992; Gray 1984; Gorecki et al. 2017; Sharma et al. 2015; Korabecny et al. 2014; Cadieux et al. 2016; de Koning et al. 2018; Castro et al. 2020).

Oxime and non-oxime reactivators are developed to remove the phosphyl moiety from the active site serine of acetylcholinesterase (AChE) (Hobbiger 1963). Such a nucleophilic attack shall restore the activity of the enzyme, enable AChE to split the neurotransmitter acetylcholine leading to a reduction or cessation of the toxic signs in OP poisoning (Eyer 2003).

In fact, since decades the standard therapy of OP poisoning includes a muscarinic antagonist, e.g. atropine, an oxime, mostly pralidoxime or obidoxime (Sidell 1974; Okumura et al. 1996; Newmark 2004; Eddleston et al. 2008; Pawar et al. 2006), and benzodiazepines as neuroprotectants and anticonvulsants (Marrs and Sellström 2007). In principle, there is no doubt that rapid administration of atropine and an oxime may be lifesaving in nerve agent poisoning (Vale et al. 2007; Eyer and Worek 2007; Sidell 1997; Thiermann et al. 2016) but the benefit of the oxime component may be limited in case of poisoning by reactivation-resistant nerve agents such as soman and tabun (Wolthuis et al. 1994; Worek and Thiermann 2013; Dawson 1994).

In contrast, a fierce debate on the value of oximes in human OP pesticide poisoning is ongoing since decades (Bajgar et al. 2007), yet on the base of rather small studies or case reports (Eddleston et al. 2002; Due 2014; Shivakumar et al. 2006; Banerjee et al. 2014; Lin et al. 2016; Tang et al. 2013; Pawar et al. 2006; Peter et al. 2006; Rahimi et al. 2006), which prompts many clinical toxicologists to refrain from using an oxime even in cases of severe OP pesticide poisoning. The reasons for the supposed failure of oximes to improve the survival rate are not fully understood but are most likely a result of mega-dose poisoning with high and long-lasting in vivo OP concentrations leading to negligible net reactivation despite oxime therapy, premature aging of the inhibited AChE due to delayed start of oxime therapy, poisoning with reactivation resistant OP, inadequate oxime dosing and premature discontinuation of oxime administration (Eyer 2003; Kharel et al. 2020). Further, coformulants of agricultural OP formulations, alcohol co-ingestion and underlying health conditions may increase OP toxicity and reduce the benefit of oxime therapy (Eddleston et al. 2009a, b; Eddleston et al. 2012).

A closer look at the research on in-service and novel reactivators in the last decade reveals a peculiar situation. Despite ongoing national and international efforts to ban highly toxic OP pesticides self-poisoning with pesticides remains a major medical problem, especially in developing countries, causing more than 100,000 deaths each year (Mew et al. 2017). However, research on reactivators is mainly focused on nerve agents and covers pesticides only marginally (Gorecki et al. 2017; Castro et al. 2020).

In this way, the last decade was characterized by the presentation of a large number of novel reactivators, the investigation of the in vitro reactivation potential; with some compounds the in vivo efficacy and the pharmacokinetic properties were assessed. This deserves a critical analysis of the potential value of novel reactivators together with a reconsideration of the concept of use of (oxime) reactivators in OP poisoning.

## The threat

Intensive research on the organic chemistry of phosphorus for more than one century resulted in the invention of various groups of organophosphorus compounds (OP), e.g. phosphates, phosphonates, phosphinates and phosphorothioates, and a countless number of structurally and toxicologically different OP (Moralev and Rozengart 2007; Timperley 2015; Worek et al. 2016c). Highly toxic OP were further developed and stockpiled for use as chemical warfare agents (Black 2016). Despite the ban of chemical warfare agents by the Chemical Weapon Convention in 1997 (United Nations Treaty Collection 1997), OP nerve agents were used repeatedly in the recent past in the Syrian Civil War since 2013 resulting in thousands of casualties and for assassinations against individuals in Malaysia 2017 and UK 2018 emphasizing the ongoing threat to the population (Costanzi et al. 2018; John et al. 2018).

The ban of highly toxic class I pesticides such as parathion or mevinphos contributed to the decrease of annual worldwide fatalities by OP pesticide self-poisoning from estimated more than 258,000 in 2007 to ~ 110,000 in 2017 (Gunnell et al. 2007; Mew et al. 2017; Bertolote et al. 2006), still an appalling high number and a continuing burden for the medical service in many countries.

**Fig. 3:**
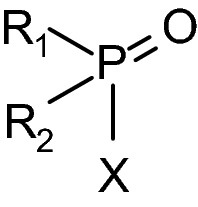
Generic structure of organophosphorus compounds with residues R_1_ and R_2_ and leaving group X. In many OP pesticides the oxygen is replaced by sulfur to reduce mammalian toxicity

A comprehensive understanding of the toxicity and toxic properties of OP is fundamental for the development of effective therapies, therapeutic concepts but also for the evaluation of limitations of antidotes and treatment regimen. There is consensus that the primary mechanism of acute OP toxicity is covalent binding of the OP to the active site of AChE resulting in AChE inhibition and development of cholinergic crisis (Jandorf et al. 1955; Holmstedt 1959; Main 1979; Sidell 2007). All OP classified as nerve agents or pesticides inhibit AChE, the potency being dependent on the agent specific inhibitory properties as a result of structural elements such as different residues bound to the central phosphorus as well as leaving groups (Fig. 3). In vitro determination of the bimolecular inhibition rate constant *k*_i_ with isolated (human) AChE enables the quantification of the inhibitory potency of specific agents and may provide an initial estimate on the toxic potential. As shown in Table 1, there is a huge range of *k*_i_ values, nerve agent and nerve agent analogs being in general more potent than pesticides with some exceptions, e.g. chlorpyrifos-oxon, the active metabolite of the pesticide chlorpyrifos, being more potent compared to the nerve agent tabun. Knowledge of these values allows an initial rough estimation of the potential in vivo toxicity. However, the actual in vivo toxicity is determined by multiple interconnected factors including volatility, chemical and biological stability, lipophilicity and finally the route of exposure (Rice 2016; Young and Watson 2020).

**Table 1:** In vitro inhibition kinetics of human AChE with selected OP

OP	*k*_i_	OP	*k*_i_
Fenamiphos	0.002	TEPP	59.7
Propophos	0.03	Methylsarin	105
Tetrachlorvinphos	0.03	Dimethyl-VE	125
Methamidophos	0.05	Leptophos	134
Monocrotophos	0.06	Tabun	182
Trichlorfon	0.07	Dimethyl-VX	222
Dicrotophos	0.15	Chlorpyrifos-oxon	269
Omethoat	0.16	Ethylsarin	327
Ethoprophos	0.23	Diisopropyl-VE	368
Heptenophos	1.38	Naled	377
Bromfenvinphos	1.43	Sarin	398
Chlorfenvinphos	1.72	VE	433
Pirimiphos-methyl-oxon	2.81	Diethyl-VX	551
Dichlorvos	3.55	VX	1150
Profenofos	4.08	n-Propylsarin	1260
Malaoxon	4.74	Soman	1930
Mevinphos	6.64	n-Butylsarin	2790
*N*-Diethyltabun	7.77	Chinese VX	3210
Dimethyl-amiton	8.57	neo-Pentylsarin	3240
Paraoxon-methyl	11.3	Cyclosarin	4390
*N*-*n*-Propyltabun	11.8	Russian VX	4580
Amiton	18.9	sec-Pentylsarin	4870
Diisopropyl-amiton	27.4	iso-Butylsarin	5330
*O*-Methyltabun	32.1	iso-Pentylsarin	5460
Paraoxon-ethyl	33.0	n-Pentylsarin	9500

Inhibitory potency of selected OP (pesticides, pesticide oxon metabolites, nerve agents and nerve agent analogues) toward human AChE in vitro. Data are from (Aurbek et al. 2006, 2010), (Worek et al. 2007a), (Bartling et al. 2007), (Worek et al. 2004, 2009) and from unpublished data. The bimolecular inhibition rate constant *k*_i_ is given as 10^5^ M^−1^ min^−1^

Vapor pressure and volatility of nerve agents are essential determinants for the key route of exposure. High volatility and water solubility qualify sarin (vapor pressure of 2.9 mmHg at 25 °C) to enter the body primarily via mucous membranes resulting in a rapid onset of toxic signs while the extremely low volatile and lipophilic VX (vapor pressure of 0.0007 mmHg at 25 °C) is of high percutaneous toxicity with a delayed onset of signs (Rice 2016; Czerwinski et al. 2006; Nozaki et al. 1995; Okumura et al. 1996).

In (suicidal) OP pesticide poisoning agent incorporation occurs mostly via the oral route and toxicity is determined by (a) cytochrome P450 mediated transformation of organophosphorothioates into the active oxon form (Buratti et al. 2003; Menzer and Dauterman 1970), (b) the agent specific inhibitory potency towards AChE causing a rapid or delayed onset of toxic signs (Thiermann et al. 1997), (c) the detoxification of the parent compound and its active metabolite by endogenous enzymes such as paraoxonase (PON1) (Furlong et al. 2000; Kaur et al. 2017) and (d) the lipophilicity as a main factor for the persistence of a specific pesticide in fat tissue and prolonged re-distribution into the systemic circulation, being of relevance with lipophilic pesticides such as parathion but less with more hydrophilic agents such as dimethoate (Eyer et al. 2009, 2003; Eyer 2003; Eddleston et al. 2008).

Chemical and biological stability, lipophilicity and route of exposure are important determinants for the toxicokinetic behavior of OP nerve agents (Benschop and de Jong 2001). Detailed studies, in part even quantifying nerve agent stereoisomers, demonstrated huge differences of toxicokinetic parameters depending on the agent and the route of exposure. Intravenous injection and inhalation exposure of sarin resulted in rapid distribution and elimination of the agent in guinea pigs with a terminal half-life of less than 60 min (Spruit et al. 2000), an even shorter terminal half-life of 23 min was determined for (−)-tabun after intravenous administration of tabun in swine (Tenberken et al. 2010). Intravenous injection of VX in guinea pigs and marmosets gave a terminal half-life between 98 and 165 min while the maximum VX concentration was reached only after 4 h in guinea pigs with percutaneous VX exposure and remained at a high level for several hours (van der Schans et al. 2003). In swine, percutaneous VX exposure again resulted in a slow increase of VX concentration in blood, a maximum at ~ 2 h followed by a plateau for at least 5 h (Reiter et al. 2011). In the end, distinct toxicokinetic properties of each individual OP have a major impact on the therapeutic regimen, i.e. duration of antidote administration and planning of medical resources.

**Fig. 4:**
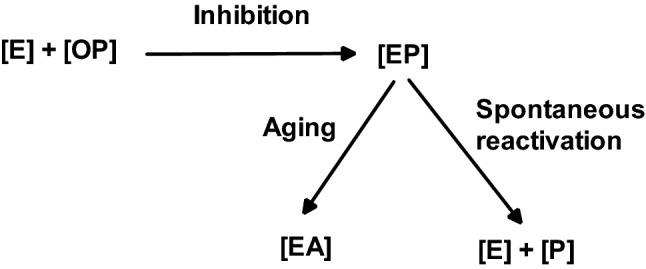
Scheme of reactions between OP and AChE [E] resulting in inhibited AChE [EP]. Post-inhibitory reactions may lead to spontaneously reactivated AChE [E] or to aged AChE [EA]

Post-inhibitory reactions of OP-inhibited AChE (Fig. 4), namely spontaneous reactivation and spontaneous dealkylation (aging), are important factors for the efficacy of, especially oxime, therapy. Again, huge differences in aging and spontaneous reactivation kinetics exist and have to be considered. Examples are aging half times of ~ 2 min with soman-inhibited AChE and almost 140 h with VR-inhibited AChE and a negligible spontaneous reactivation with G-agent inhibited AChE while AChE inhibited by a dimethyl-OP, e.g. malaoxon, exhibits a rapid spontaneous reactivation with a t½ of 0.7 h (Worek and Thiermann 2013). Hence, soman poisoning will lead to aged AChE and ineffective oxime therapy while in a moderate poisoning with dimethyl-OP (e.g. malathion, dimethoate) spontaneous reactivation of the inhibited AChE may contribute to the oxime effect.

Consequently, OP pesticides and nerve agents have a common mechanism of action, i.e. covalent binding to AChE and inhibition of its physiological function, but depending on the structure and resultant physico-chemical and biological properties a large variability of toxic effects and inherent susceptibility towards oxime therapy has to be taken into account (Table 2). Hence, the large amount of influencing variables needs to be considered for the development of optimized therapies.

**Table 2:** Impact of differential toxic properties of OP pesticides and nerve agents on oxime therapy

OP subclass	OP	Primary route of exposure	Onset of toxic signs	Agent persistence	Oxime efficacy (AChE reactivation)^a^	Duration of (oxime) therapy
Nerve agents	Sarin	Inhalation	Rapid (min)	Low	Rapid	Initial emergency administration
	Tabun	Inhalation	Rapid (min)	Low	Slow, partial	
	Soman	Inhalation	Rapid (min)	Low	Absent (aging)	
	VX	Percutaneous	Delayed (h)	High	Rapid	Prolonged (days)
Pesticides	Diazinon (parathion)	Oral (suicide)	Rapid (min)	Moderate to high	Rapid	Prolonged (days) in case of high OP body load
	Malathion, dimethoate	Oral (suicide)	Delayed (h)	Moderate to high	Rapid, may be absent due to rapid aging	Prolonged (days) in case of high OP body load and incomplete aging
	Profenofos	Oral (suicide)	Delayed (h)	Unknown	Absent (aging)	Initial emergency administration

^a^Extent of net AChE reactivation will strongly depend on the incorporated OP dose

## The oxime concept

### General aspects

Treatment of OP poisoning with atropine and an oxime is the general procedure since the implementation of 2-PAM in the 1950s and obidoxime in the 1960s into clinical use (Eyer 2003; Jokanovic 2009; Worek and Thiermann 2013; Eddleston et al. 2002). There is no doubt that antimuscarinics, primarily atropine, are essential to counteract OP-induced cholinergic overstimulation at muscarinic receptors and may resolve severe toxic signs such as central nervous respiratory depression, bradycardia, bronchoconstriction and bronchorrhoe (McDonough and Shih 2007). The reversible muscarine receptor antagonist atropine may be considered as a generic antidote being effective against all OP, major disadvantages are the solely symptomatic effect and the inability to counteract OP effects on nicotinic receptors. In case of sufficient reactivation of inhibited AChE the life threatening peripheral respiratory block, mediated by nicotinic receptors, can be resolved. This was the primary reason to introduce nucleophilic oximes as reactivators of inhibited AChE at nicotinic, but also at muscarinic, synapses.

**Fig. 5:**
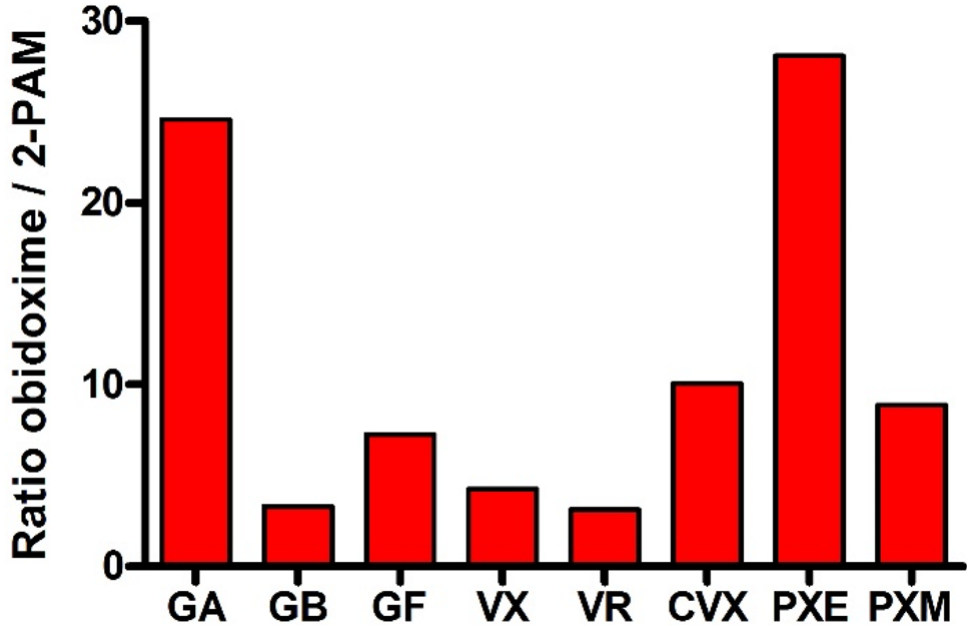
Ratio of bimolecular reactivation rate constants *k*_r2_ of obidoxime and 2-PAM for GA tabun, GB sarin, GF cyclosarin, VX, VR Russian VX, CVX Chinese VX, PXE paraoxon-ethyl and PXM paraoxon-methyl

Already at the beginning of the oxime era, experimental and clinical data indicated that oximes have a limited effect under various circumstances (Hobbiger 1963). It turned out that the available oximes 2-PAM and obidoxime reactivate AChE inhibited by different OP to various extent, fail to reactivate aged AChE and have an uncertain therapeutic effect in human OP pesticide poisoning (Namba 1971; Willems et al. 1971; Erdmann 1968; Zech et al. 1967; Loomis and Salafsky 1963; Heilbronn and Tolagen 1965). Hereby, obidoxime exhibits a markedly higher reactivating potency compared to 2-PAM with a variety of nerve agents and pesticides (Fig. 5) but also fails to reactivate soman-inhibited AChE (Wolthuis et al. 1994). Numerous oximes were synthesized in the following decades, these research efforts have been reviewed extensively and shall not be addressed further (cf. Bismuth et al. 1992; Dawson 1994; Kassa 2002; Reiner and Simeon-Rudolf 2006; Eyer and Worek 2007; Worek and Thiermann 2013).

At present, pralidoxime (2-PAM, P2S) is the most widely used oxime while obidoxime and TMB-4 are limited to a few countries (Worek and Thiermann 2013). Only two bispyridinium oximes, MMB-4 and HI-6, synthetized in 1959 and 1968, respectively (Hobbiger and Sadler 1959; Stark 1968), were transferred to advanced development and are considered as potential replacement of 2-PAM for the treatment of nerve agent poisoning (Lundy et al. 2011). Many promising oximes never reached this stadium, the reasons, apart from limited funding, being widely unknown. The HI-6 analog HLö 7, bearing two oxime functions in position 2 and 4 at one pyridinium ring, was considered to be a broad spectrum oxime with superior reactivating potency compared to HI-6 and good therapeutic efficacy against different nerve agents including tabun (Eyer et al. 1992; Lundy et al. 1992). A higher toxicity compared to HI-6, low stability in aqueous solutions and a challenging synthesis were the most likely reasons for the limited interest into HLö 7 in the recent years and the focus on HI-6 as the prime candidate oxime (Psotka et al. 2017; Hsu et al. 2019).

The peculiar situation of having thousands of experimental oximes, only five oximes, developed between 1955 and 1968, in clinical use or advanced development but an ongoing need for a better reactivator deserves a closer and critical look on the oxime concept and on factors influencing the in vivo efficacy of oximes.

### Theoretical and kinetic considerations

**Fig. 6:**
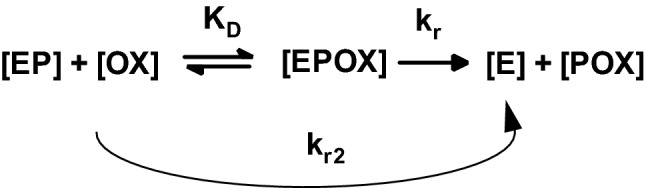
Reaction scheme for the reactivation of OP-inhibited AChE by oximes. [E] native AChE; [EP] OP-inhibited AChE; [OX] oxime; [EPOX] Michaelis complex; [POX] phosphyloxime;* K*_D_ dissociation constant; *k*_r_ reactivity constant; *k*_r2_ bimolecular reactivation rate constant

Reactivation of OP-inhibited AChE is the primary mechanism of action of oximes. Additionally, there are speculations about postulated direct pharmacological effects of oximes. Such effects, namely improved survival in soman poisoning in the absence of AChE reactivation, could be shown in various animal models but there is no evidence in humans (van Helden et al. 1996; Seeger et al. 2011). According to the generally accepted reaction scheme (Fig. 6) the reactivation is determined by the affinity and reactivity of an oxime towards the OP-inhibited AChE which can be quantified in vitro by the dissociation constant *K*_D_ and the reactivity constant *k*_r_. These parameters were determined in numerous studies using AChE from different origin, different experimental protocols and different methods of calculation (Miller et al. 1984; Hrvat et al. 2018) resulting in a wide range of numbers even for a specific OP-oxime combination (Worek and Thiermann 2013).

In vitro reactivation constants allow model calculations to estimate minimum *k*_r_ and *K*_D_ values for achieving a sufficient reactivation level and to calculate necessary oxime concentrations to reach a certain reactivation level after a defined time (Worek et al. 2011b). From these calculations a reactivity constant *k*_r_ of > 0.1 min^−1^ and a dissociation constant of *K*_D_ < 100 µM were proposed. Hereby, a *k*_r_ as high as possible is desirable since it determines the reactivation half-time. An extremely high affinity of an oxime, i.e. very low *K*_D_, may lead to negative effects due to an oxime-induced inhibition of native or reactivated AChE which may limit the tolerable concentration in vivo (Wille et al. 2010; Worek et al. 2012; Kovarik et al. 2010).

It has to be considered that in vitro determination of reactivation kinetics does not adequately resemble the in vivo situation. Purified AChE is inhibited by a defined OP concentration and excess inhibitor is removed. Then, OP-inhibited AChE is incubated with a constant oxime concentration for a defined time in the absence of substrate and potential side reactions which may be present in vivo are excluded. Otherwise, side reactions such as re-inhibition of reactivated AChE by phosphyloxime formed during the reactivation process may affect in vitro assays but was not verified in vivo (Ashani et al. 2003; Eyer and Worek 2007).

**Fig. 7:**
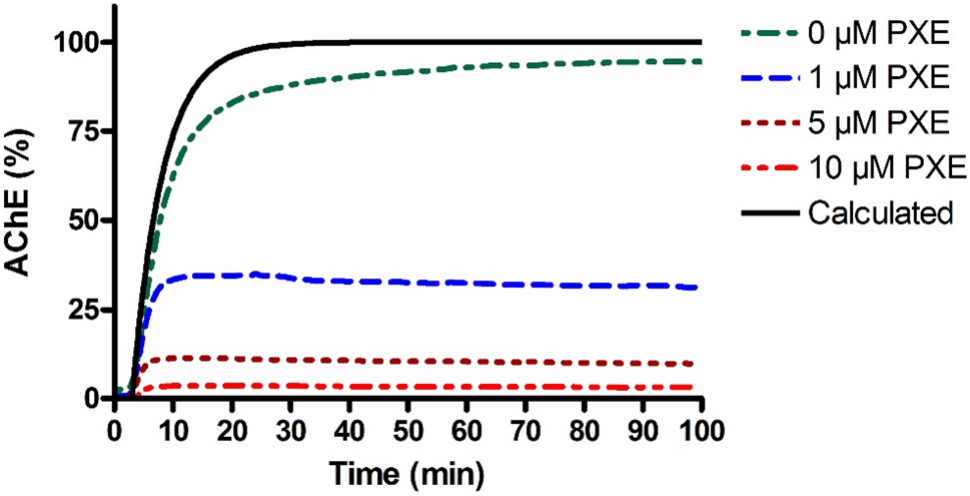
Reactivation of paraoxon-inhibited human AChE by obidoxime (10 µM) in the presence of paraoxon (0–10 µM) in a dynamic model with online recording of AChE activity (Worek et al. 2015). Calculation gives the theoretical reactivation based on in vitro reactivation constants (Worek et al. 2011a)

An even greater impact on oxime-induced reactivation has the presence of OP in vivo. This is determined by the aforementioned physico-chemical properties, exposure route and dose. The effect of OP concentration can be simulated by theoretical calculations based on kinetic constants and pharmaco- and toxicokinetic parameters (Worek et al. 2005) and in appropriate in vitro models. Figure 7 demonstrates the effect of different paraoxon concentrations (0–10 µM) on the net reactivation of inhibited AChE by a therapeutic obidoxime concentration (10 µM) in a dynamic in vitro model with online determination of AChE activity. Reactivation of pre-inhibited AChE by obidoxime in the absence of excess paraoxon resulted in an almost complete reactivation being very comparable to calculated activities based on reactivation constants. In the presence of 1 µM paraoxon, a concentration indicating a severe suicidal parathion poisoning (Eyer et al. 2003), the net reactivation was already reduced to some 30% and was only marginal at higher paraoxon concentrations.

Percutaneous poisoning of guinea pigs with VX resulted in long-lasting, toxicologically relevant VX concentrations in blood (Joosen et al. 2010). A single injection of a high obidoxime dose (24.6 mg/kg i.m.) resulted in an initial increase of AChE activity followed by a constant decrease due to the short obidoxime plasma half-live. Repeated injections of a lower obidoxime dose (8.2 mg/kg i.m.) preserved an at least partial AChE activity underlining the need to adopt the treatment regimen to the individual case. A thorough analysis of clinical cases demonstrated the decisive impact of the OP concentration on net AChE reactivation in patients treated by obidoxime (Eyer et al. 2003, 2009; Thiermann et al. 2009). In the end, an oxime with high reactivating potency such as obidoxime with diethyl-OP- or VX-inhibited AChE may fail to achieve sufficient net reactivation in vivo due to persisting, toxicologically relevant OP concentrations.

**Fig. 8:**
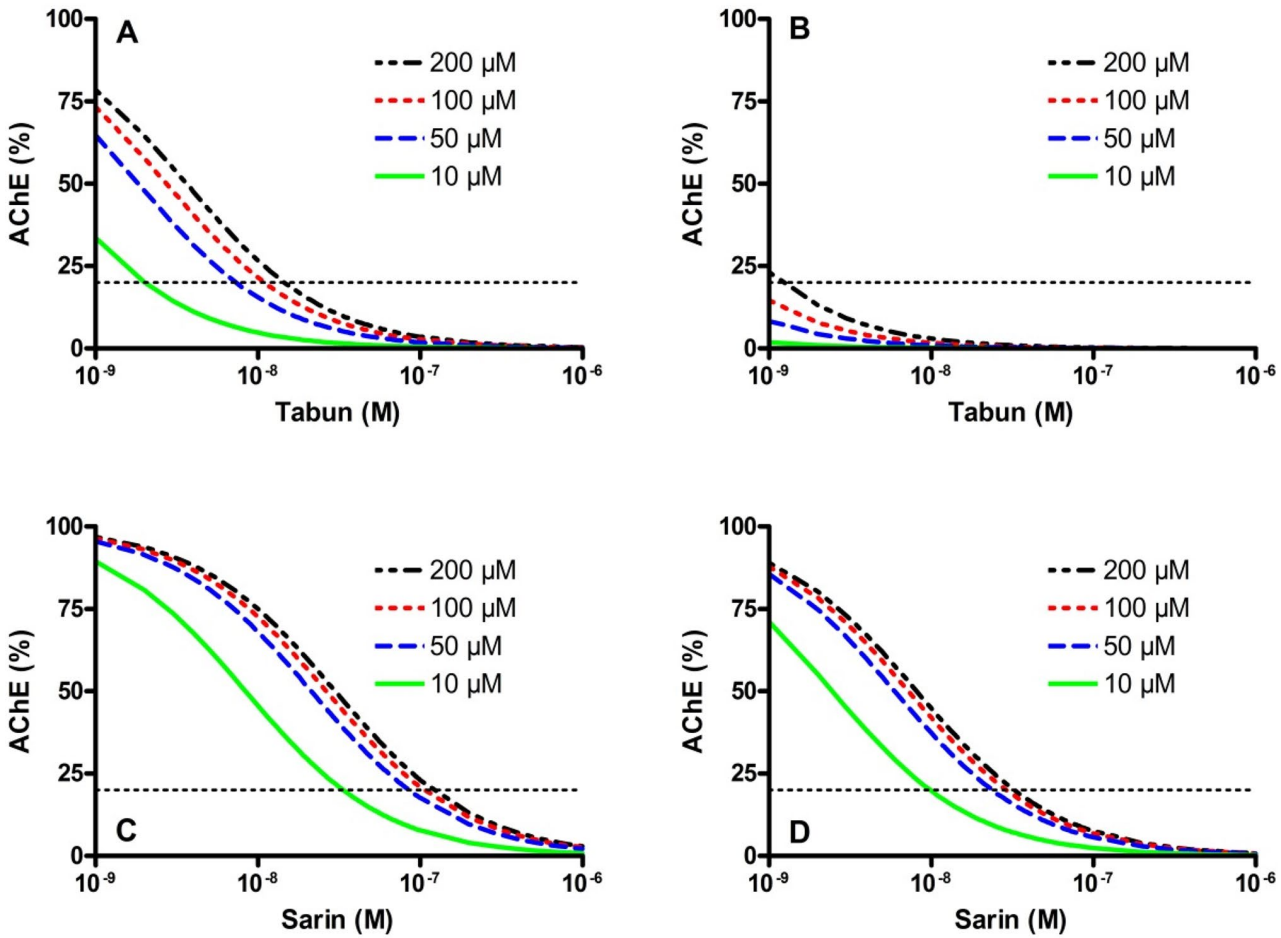
Calculated steady-state AChE activities in the presence of tabun (**a**, **b**) or sarin (**c**, **d**) and obidoxime (**a**, **c**) or 2-PAM (**b**, **d**). Calculations are based on experimental reactivation constants of obidoxime (tabun: *k*_r_ 0.04 min^−1^, *K*_D_ 97.3 µM; sarin: *k*_r_ 0.937 min^−1^, *K*_D_ 31.3 µM) and 2-PAM (tabun: *k*_r_ 0.01 min^−1^, *K*_D_ 695 µM; sarin: *k*_r_ 0.25 min^−1^, *K*_D_ 27.6 µM) and the bimolecular inhibition rate constants *k*_i_ of tabun (7.4 × 10^6^ M^−1^ min^−1^) and sarin (2.7 × 10^7^ M^−1^ min^−1^) (Worek et al. 2004) and were performed for oxime concentration of 10–200 µM. The dotted horizontal line resembles the cutoff AChE activity (20%). For calculation the equation [E]/[EP + EPOX] = *k*_r_/(*k*_i_ × [OP] × (1 + *K*_D_/[OX])) was applied (Eyer 2003)

Eyer and co-workers presented models for the calculation of steady-state AChE activities in the presence of different OP and oxime concentrations and for the estimation of oxime concentrations necessary to achieve a defined level of AChE reactivation (Thiermann et al. 1999; Eyer 2003; Worek et al. 2011b, 2016b). Here, the agent-specific reactivating potency of an oxime and the inhibitory potency of an OP are decisive for the required therapeutic oxime concentration, indicating that the necessary oxime concentration will vary for different OP. Figure 8 exemplifies the effect of increasing tabun and sarin concentrations on the reactivation of AChE by obidoxime and 2-PAM. Due to the differential reactivating potency of these oximes recommended therapeutic concentrations, i.e. 10 µM obidoxime, initially ~ 30 µM pralidoxime, now the proposed target concentration is 100 µM pralidoxime (Thiermann et al. 1999; Eddleston et al. 2009a, b; Sundwall 1961), will reach a cut-off AChE activity only at low nanomolar tabun concentrations (obidoxime) or even fail (2-PAM). Due to the substantially higher reactivating potency of both oximes with sarin-inhibited AChE obidoxime and 2-PAM should reach the minimum AChE level even at sarin concentrations up to some 50 nM. Hence, this example underlines the need to analyze the potential oxime-induced reactivation separately for each oxime-OP combination to assess the potential value of a specific reactivator.

Finally, the selection of the AChE source has a major impact on the evaluation of oxime potency and eventually efficacy. Various studies demonstrated in part significant and hardly predictable differences in the reactivation kinetics depending on the species (human vs animal AChE), OP and oxime (Luo et al. 2005, 2007; Worek et al. 2002, 2010, 2011a).

### Impact of oxime toxicity

Therapeutically necessary oxime concentrations may not be tolerable due to the intrinsic toxicity of oximes (Calesnick et al. 1967; Marrs 1991). Multiple studies demonstrated a broad range of in vitro, i.e. AChE inhibition, and animal in vivo toxicity of established and experimental oximes ranging from > 800 mg/kg (HI-6) down to ~ 2 mg/kg (K108) after i.m. administration in different species (Marrs 1991; Kassa 2002; Reiner and Simeon-Rudolf 2006; Lorke and Petroianu 2009; Musilek et al. 2010). Unfortunately, relevant toxicity data are missing for most of the experimental oximes.

Data on toxicity of oximes in non-poisoned humans are sparse and are mainly a by-product of pharmacokinetic investigations. Only 2-PAM (P2S), TMB-4, obidoxime and HI-6 were tested in humans at doses related to the assumed therapeutic dose and only mild to moderate side effects (circulatory, gastrointestinal, sensory) were observed, TMB-4 being considered to be the most toxic of these oximes (Calesnick et al. 1967; Wiezorek et al. 1968; Sidell and Groff 1970; Erdmann et al. 1965; Holland and Parkes 1976; Xue et al. 1985; Clement et al. 1995).

Most of these human volunteer studies administered parenteral oxime doses recommended as initial dose for treatment of OP patients, i.e. ~ 2 g 2-PAM (Eddleston et al. 2004; Newmark 2007), 250 mg obidoxime (Erdmann and Clarmann 1963) and 500 mg HI-6 (Kusic et al. 1991). Calesnick et al. injected up to 45 mg/kg 2-PAM chloride i.m. or i.v. (Calesnick et al. 1967), Wiezorek et al. up to 1400 mg 2-PAM iodide (Wiezorek et al. 1968), Holland and Parkes up to 23.5 mg/kg P2S i.m. (Holland and Parkes 1976), Kusic et al. and Clement et al. up to 500 mg HI-6 dichloride i.m. (Kusic et al. 1985; Clement et al. 1995).

The situation is slightly different with obidoxime and TMB-4. Sidell and Groff injected up to 10 mg/kg obidoxime i.m. (~ 700 mg) and got a *C*_max_ of almost 140 µM in plasma, exceeding the recommended therapeutic concentration more than tenfold (Sidell and Groff 1970). Only a transient increase in blood pressure and heart rate, generalized warmth and numbness of the facial area were reported. At present, Israel uses a combination autoinjector with 80 mg TMB-4 (Bentur et al. 2006). Two volunteer studies used substantially higher doses and recorded only mild adverse effects. The intravenous injection of 800 mg TMB-4 resulted in a slight, transient increase in heart rate and decrease in blood pressure, generalized warmth, numbness of the facial area and dizziness (Wiezorek et al. 1968). TMB-4, up to 30 mg/kg i.v. or i.m. (~ 2100 mg) resulting in a maximum concentration of ~ 200 µM, induced a delayed hypotension, other adverse effects were not reported (Calesnick et al. 1967). These data indicate that higher than the recommended initial obidoxime or TMB-4 doses could be tolerated in healthy volunteers while the available human data do not allow an estimation of the maximum tolerable 2-PAM and HI-6 dose.

Although oximes were tolerated well in healthy volunteers, limited case reports hypothesize that the oximes 2-PAM and obidoxime may have unexpected heart effects in OP poisoned patients. In two cases with suicidal OP poisoning heart arrest was attributed to administration of 2-PAM (Scott 1986; Jeong et al. 2015) and Finkelstein and coworkers observed a high frequency of cardiac arrhythmias in patients treated with high dose obidoxime (Finkelstein et al. 1989). However, these findings are not suited for a direct connection of oximes and pathological cardiac effects. It has to be mentioned that OP poisoning may result in severe effects on the cardiovascular system and adverse effects of other drugs such as atropine have to be considered. In fact, multiple studies demonstrated a variety of cardiac disturbances, e.g. prolonged QTc time, ventricular arrhythmia and Torsades de Pointes, in patients treated exclusively with atropine (Kiss and Fazekas 1979; Ludomirsky et al. 1982; Brill et al. 1984; Vijayakumar et al. 2011). In conclusion, a proper assessment of the toxicity of novel reactivators is essential and potential differences between healthy volunteers and OP poisoned patients have to be considered in the development and selection of new oximes.

### Interim résumé

To this end, oximes are the only therapeutic option to overcome the toxic effects of OP nerve agents and pesticides. Alternative approaches such as prevention of toxic OP concentrations in target tissues by stoichiometric and catalytic (bio)scavenger or antagonizing of downstream effects of acetylcholine at nicotinic receptors, e.g. by antinicotinics, are in an (early) experimental stage or not yet available as a licensed drug (Masson 2016; Tattersall 2016; Seeger et al. 2011; Niessen et al. 2018). Under ideal conditions oximes may ensure survival by restoring neuromuscular transmission and may reverse other toxic effects, probably except of the CNS, in the early phase of OP poisoning. However, irrespective of the kinetic properties of an oxime the success of (ongoing) oxime therapy will strongly rely on additional factors, OP properties and dose, adequate atropine dosing, administration of anticonvulsants, rapid whole body decontamination in case of percutaneous exposure, rapid gastric lavage after oral uptake and eventually intensive care treatment including artificial ventilation. Hence, oximes cannot be considered as an independent treatment but are always part of a holistic therapeutic concept.

## Novel reactivators

### Oxime reactivators

In the past 10–15 years various research groups presented novel reactivators, primarily oximes (e.g. Fig. 2, compounds 1–6) but more recently also non-oxime reactivators (e.g. Fig. 2, compounds 7 and 8). This research was directed to identify more effective broad spectrum reactivators and compounds which easily penetrate the blood–brain-barrier. Additional research is focused on reactivators with improved reactivating potency towards OP-inhibited butyrylcholinesterase (BChE) as a prerequisite to enable multiple reactivation—inhibition cycles of this enzyme and to reduce the OP concentration in blood more rapidly, these efforts were reviewed repeatedly and shall not be discussed in detail (cf. Kuca et al. 2006; Musilek et al. 2011; Mercey et al. 2012b; Korabecny et al. 2014; Sharma et al. 2015; Masson and Nachon 2017; Gorecki et al. 2017; Taylor et al. 2019; Kobrlova et al. 2019; Chambers and Meek 2020; Castro et al. 2020).

Some novel reactivators were considered promising by their inventors and deserve a more detailed evaluation. The bispyridinium oxime K203, [(*E*)-1-(4-carbamoylpyridinium)-4-(4-hydroxyiminomethylpyridinium)-but-2-ene dibromide], was initially considered as a universal reactivator of nerve agent-inhibited AChE (Kuca et al. 2015). However, in vitro tests with rat brain AChE revealed only a moderate reactivation of tabun-, sarin- and VX-inhibited AChE using an extraordinary high K203 concentration (1 mM) but virtually no activity against cyclosarin and Russian VX. In later studies, K203 was proposed as a superior reactivator of tabun-inhibited AChE and in fact it turned out that K203 was a better reactivator compared to obidoxime but still too weak with a second order reactivation rate constant of 0.92 mM^−1^ min^−1^ (Kuca et al. 2018; Gorecki et al. 2019; Winter et al. 2016).

Sit et al. presented a large series of uncharged, zwitterionic oximes aimed to provide centrally acting reactivators (Sit et al. 2011). Among these, RS194B (Fig. 2, compound 4) was considered as new lead compound but turned out to be a weak reactivator of paraoxon-, sarin-, cyclosarin-, VX- and tabun-inhibited human AChE and in most cases substantially less potent compared to 2-PAM (Radic et al. 2012; Kovarik et al. 2013; Sit et al. 2018). Post-exposure therapy of OP poisoned mice by RS194B (125 mg/kg) and atropine (10 mg/kg) provided good protective ratios for paraoxon, sarin and VX (unfortunately, the protective ratios of atropine alone therapy were not provided for comparison) but had no effect with soman and tabun (Radic et al. 2012). In mice exposed to 0.75 LD_50_ sarin or VX s.c. 125 mg/kg RS194B i.m. resulted only in a partial reactivation of blood AChE in VX poisoned mice which raises the question whether the therapeutic effect of this oxime is related to AChE reactivation (Sit et al. 2018). In guinea pigs challenged with 0.85 LD_50_ nerve agents or pesticides RS194B (126 mg/kg in combination with 0.4 mg/kg atropine) had no convincing therapeutic effect (Wilhelm et al. 2014). In contrast, RS194B treatment of macaques after inhalation exposure to sarin and paraoxon resulted in substantial AChE reactivation and clinical improvement which may indicate substantial species differences in the effect of this oxime (Rosenberg et al. 2017, 2018).

French research groups presented a larger number of uncharged oximes which were in general less potent compared to the lead oximes obidoxime and HI-6 (Mercey et al. 2012a, b; Renou et al. 2013, 2014; Saint-Andre et al. 2011; Kliachyna et al. 2014; Zorbaz et al. 2018; Santoni et al. 2018). Exceptions are shown in Fig. 2 with compounds 5 and 6 having a comparable and in part higher reactivating potency compared to obidoxime and HI-6 (Mercey et al. 2012a; Santoni et al. 2018). With compound 6 s order reactivation constants > 10 mM^−1^ min^−1^ were determined with paraoxon-, sarin-, VX- and even tabun-inhibited human AChE. Unfortunately the use of this oxime will be most likely limited by a high intrinsic inhibitory potency towards native human AChE (IC_50_ 2.3 µM).

In general, there is a huge difference in the reactivating potency of oximes towards OP-inhibited human AChE and BChE, BChE being substantially more reactivation resistant (Aurbek et al. 2009). In consequence a further line of research is directed to the identification of oxime structures with markedly improved reactivating potency towards OP-inhibited human BChE with the ultimate goal to provide a system where plasma and tissue BChE is transferred to a pseudo-catalytic scavenger (Radic et al. 2013). Using different scaffolds charged and uncharged oximes were presented and showed in part a remarkable improvement in comparison to standard oximes (Sit et al. 2014; Katalinic et al. 2016; Zorbaz et al. 2019; Malinak et al. 2020). This approach is presumably of low relevance in case of poisoning by OP with short residence time such as sarin but may be beneficial in intoxications by persistent OP such as VX or lipophilic pesticides. Further studies are needed to investigate in detail potentially effective and tolerable concentrations and to assess whether the repeated reactivation of low, physiological BChE concentrations may result in a significant reduction of an OP concentration in the body. This is an important question which may be illustrated by a simplified calculation. Assuming a percutaneous exposure by 10 mg VX, resorption of 50% agent and a blood volume of 5 L would result in a concentration of ~ 3700 nmol/l VX. By taking a plasma BChE concentration of ~ 50 nmol/l (Nachon et al. 2013) and using an oxime with a rather rapid reactivation half-time of 1 min it may take up to 150 min to detoxify the agent. This example neglects dynamic processes such as ongoing agent resorption after percutaneous exposure, distribution of agent into tissues and rapid clearance of the oxime after bolus injection but may point to the fact that the concept of pseudo-catalytic scavenging will require highly reactive BChE reactivators which are not available at present, will at best reduce the residence time of an OP but will not prevent severe toxic OP effects.

### Non-oxime reactivators

A recent approach is the search for non-oxime reactivators as an alternative to oxime-based reactivators. First, Katz and coworkers investigated the ability of the antimalarial drug amodiaquine and its analog ADOC (Fig. 2, compound 7) to reactivate paraoxon-, sarin- and DFP-inhibited human AChE and demonstrated reactivation by ADOC being superior compared to 2-PAM for all three OP (Katz et al. 2015). Later, Bierwisch et al. found partial reactivation of VX-inhibited human AChE by amodiaquine but also pointed to the limitation of high intrinsic inhibitory potency of this compound (Bierwisch et al. 2016). The reactivating potential of ADOC was verified with nerve agent-inhibited human AChE and selected ADOC analogues exhibited at least some reactivating potential (Cadieux et al. 2016). Different ADOC derivatives were synthetized by de Koning et al. and the Mannich phenol PADOC (Fig. 2, compound 8) demonstrated a good reactivating effect towards paraoxon-, sarin-, cyclosarin- and VX-inhibited human AChE but failed with tabun-inhibited AChE (de Koning et al. 2018). The reactivating potency of PADOC was higher (paraoxon), comparable (VX) and lower (sarin, cyclosarin) compared to HI-6 (Horn et al. 2018). So far no convincing structure–activity-relationship could be derived for the available ADOC derivatives but there seems to be a relationship between the ability to reactivate and the affinity towards native AChE which limits the maximum compound concentrations in vitro and potentially in vivo. Further research on non-oximes should not be limited to ADOC derivatives but should investigate a broader spectrum of scaffolds (Bhattacharjee et al. 2012, 2015).

## The challenging oxime development

The discovery of pralidoxime (2-PAM) in 1955 (Childs et al. 1955; Wilson and Ginsburg 1955) and rapid implementation into clinical use (Namba and Hiraki 1958) was a milestone in the development of more effective therapies of OP poisoning. Surprisingly, this oxime, as well as obidoxime, was licensed in many countries for use in OP poisoning despite of a limited agent spectrum, unspecified therapeutic concentration and largely unknown toxicity; it is hardly conceivable that these oximes would get a license under present regulations having only the dataset of the late 1950s and early 1960s. Extensive research was initiated in various Western countries and led to the synthesis of a countless number of oximes until end of the 1980s. Interest in new oximes increased again some 10–15 years ago, again leading to the publication of a huge number of oximes and more recently of non-oxime reactivators. Despite the discovery of thousands of compounds only five oximes, 2-PAM, obidoxime, HI-6, TMB-4 and MMB-4, synthesized between 1955 and 1968, are in use by civilian and military medical services or in advanced development (Worek and Thiermann 2013). The cause of this peculiar situation needs further consideration since it may impact the advanced development and transfer into clinical use of promising, new reactivators.

In the past six decades oxime research and development was primarily funded by military organizations and was focused on improved therapies against nerve agents, primarily soman and tabun. The emphasis was put on the initial emergency treatment of military personnel by autoinjectors and less on the follow-up therapy in military or civilian medical facilities. Accordingly, a huge number of studies on oxime effectiveness were performed using human autoinjector equivalents, thereby neglecting species dependence. Moreover, Hamilton and Lundy pointed to the fact that the intended use in a military community should ease the licensing of a new oxime since there is no need to consider risk groups such as children, elderly and pregnant women (Hamilton and Lundy 2007). On the other hand, the focus on military use limits the procurement quantity and is not attractive for the pharmaceutical industry. In consequence, advanced development and licensing is performed by military organizations which may be one reason for the long-lasting process to develop new autoinjectors with HI-6 or MMB-4 as active ingredients (Lundy et al. 2011; Harvilchuck et al. 2013). In fact, the program for the development of a HI-6 autoinjector started more than 20 years ago in several European countries and Canada and of a MMB-4 autoinjector more than a decade ago in the USA, both efforts are still in progress.

The selection and implementation of new and more effective oximes for treatment of OP pesticide poisoning faces different problems. Most intentional OP pesticide intoxications occur in developing countries having limited financial resources (Mew et al. 2017; Eddleston 2019). Despite a huge number of patients requiring effective medical treatment the ongoing controversy on the virtue of oximes (Blumenberg et al. 2018; Kharel et al. 2020), the difficulty in designing meaningful phase II clinical studies (Eddleston 2019) and the reluctance of the pharmaceutical industry to engage in oximes hampers a broader and optimized use of the established oximes 2-PAM and obidoxime and makes intensified research on improved reactivators against OP pesticides unlikely.

## Do we need better (oxime) reactivators?

The “big five”, 2-PAM, obidoxime, HI-6, TMB-4 and MMB-4, are afflicted with numerous disadvantages. Agent specific reactivating potency, limited agent spectrum, poor blood–brain-barrier penetration and limited stability are major issues and call for more effective reactivators.

Yes, we need better reactivators but the presently available database on reactivators presented in the past few years gives no indication of a candidate which is clearly superior to the classical oximes, especially to obidoxime and HI-6. One reason for this judgement is the lack of sufficient experimental data. A summary of available reactivation constants of selected oximes is shown in Table 3. It exemplifies that even for these compounds, being considered by the authors as promising or even lead compounds, no complete data set on in vitro reactivation is available.

**Table 3:** Second order reactivation rate constants of selected novel oximes

Oxime	GA	GB	GF	VX	VR	PXE	MAL
K203^a^	0.93	n.a	0.5	n.a	n.a	3.6	n.a
RS194B	0.001^b^	1.3^c^	0.23^c^	1.6^c^	n.a	0.05^c^	n.a
Compound 5^d^	1.7	n.a	n.a	20	n.a	19	n.a
Compound 6^e^	11.5	12.2	n.a	13.6	n.a	19.2	n.a

Second order reactivation rate constants are given as mM^−1^ min^−1^. Data are from ^a^(Winter et al. 2016), ^b^(Kovarik et al. 2013), ^c^(Sit et al. 2018), ^d^(Mercey et al. 2012a), ^e^(Santoni et al. 2018). GA tabun; GB sarin; GF cyclosarin; VR Russian VX; PXE paraoxon; MAL malaoxon. n.a. not available

In the end, limited resources require a structured, step-wise approach and a comprehensive set of in vitro and in vivo studies for the successful identification and down-selection of candidate reactivators (Table 4). In vitro reactivation kinetics with human and animal AChE (and BChE) using a broad spectrum of OP is the basis for the initial selection of promising compounds, for the evaluation of potential species differences and for the estimation of therapeutic concentrations. In vivo efficacy studies, again using different animal species, multiple OP with different route of exposure, different treatment regimen and extensive monitoring of physiological, biochemical and clinical parameters as well as OP and oxime concentrations will allow a sound assessment of the therapeutic value of candidate reactivator(s) in experimental animals and will be the base for an initial extrapolation to humans. Additional studies such as pharmacokinetics, safety pharmacology and toxicity are mandatory to finalize the preclinical phase.

**Table 4:** Requirements for the investigation and down-selection of candidate oximes

Study phase	Keystones	Study types (examples)
Preclinical phase	In vitro reactivation kinetics	Human and animal AChE/BChE; multiple OP; species differences
	Ex vivo pharmacodynamics	Isolated organs (e.g. diaphragm)
	In vivo efficacy	Different animal species (guinea pig, swine, NHP); multiple OP; different route of OP exposure; single, multiple oxime injections; various oxime concentrations; different adjuncts; physiological, biochemical, behavioral monitoring; OP & oxime concentrations; AChE & BChE activities
	In vitro pharmacokinetics	Cell culture; isolated organs (metabolism, blood–brain-barrier penetration)
	In vivo pharmacokinetics	Different animal species (guinea pig, swine, NHP); single, multiple oxime injections
	In vitro toxicity	Human and animal AChE / BChE (inhibition); cell culture (e.g. cytotoxicity, mutagenicity, carcinogenicity)
	In vivo toxicity	Different animal species (acute, subchronic, chronic toxicity)
	Safety pharmacology	Central nervous, cardiovascular, respiratory, gastrointestinal and renal system
	Drug interactions	In vitro and in vivo interactions with adjuncts (atropine, anticonvulsants)
Clinical phase	Phase I	Safety screening; adverse effects; pharmacokinetics (oxime alone and in combination with other antidotes)
	Phase II	Case reports; case series; randomized clinical trials (OP pesticides)

Phase I clinical studies are an essential component in the licensing process but human efficacy studies (Phase II) will be a specific challenge. For obvious ethical reasons it will never be possible to expose humans intentionally to nerve agents or pesticides. Hence, the way out could be the use of specific regulations for medical countermeasure drugs such as the US Federal Drug Administration animal rule (Aebersold 2012). Alternatively, one could envisage to test candidate reactivators in OP pesticide poisoned patients. This would only be ethical if such a compound has convincing reactivating potency towards AChE inhibited by anticipated pesticides and would require a careful preselection of patients, e.g. exclusion of patients with mega dose poisoning or premature aging, i.e. a too long time-span between resorption and initial onset of oxime administration, to demonstrate the therapeutic efficacy.

Most likely it will not be possible to identify a single reactivator fulfilling all requirements, e.g. broad spectrum efficacy and blood–brain-barrier penetration. Combining two or more reactivators with an overlapping spectrum and desired properties could be an alternative or interim solution (Kovacevic et al. 1991). Various in vitro and in vivo studies demonstrated a beneficial effect of combination of appropriate oximes such as obidoxime and HI-6 or TMB-4 and HI-6 primarily by broadening the spectrum (Maksimovic and Kovacevic 1989; Clement et al. 1987; Kassa et al. 2010, 2011a, b; Caisberger et al. 2018; Worek et al. 2007b, 2016a). This approach could be considered with (future) experimental reactivators by combining compound(s) with high reactivating potency and improved blood–brain-barrier penetration.

## Conclusion and outlook

Despite long-lasting and extensive research on alternative therapies such as (bio)scavengers, oximes will remain a vital component for the treatment of OP poisoning. The oximes in use (“big five”) have well-known limitations and more effective reactivators being superior against a broad spectrum of OP nerve agents and pesticides, showing an improved blood–brain-barrier penetration and potentially offering the option to transform blood and tissue AChE and BChE into a pseudo-catalytic scavenger are needed. Up to now, none of the countless experimental oximes and non-oxime reactivators exhibit superior properties being suitable as replacement of established oximes.

Investigation of future reactivators should include standard nerve agents, nerve agent analogs, novel agents and pesticides to evaluate the potential and the limitations for an as broad as possible agent spectrum. Identifying a reactivator or combination of reactivators with sufficient efficacy against all potential OP threat agents and conceivable scenarios should be the ultimate goal; however, its realization is not foreseeable.

Advanced development, licensing and procurement of candidate reactivators will depend on available resources and, in view of the experiences with HI-6 and MMB-4, will most likely be a longsome process. Hence, an interim solution could be the combination of established oximes with a complimentary spectrum such as obidoxime and HI-6 for the treatment of nerve agent poisoning.
